# A versatile microsatellite instability reporter system in human cells

**DOI:** 10.1093/nar/gkt615

**Published:** 2013-07-16

**Authors:** Wouter Koole, Henning S. Schäfer, Reuven Agami, Gijs van Haaften, Marcel Tijsterman

**Affiliations:** ^1^Department of Toxicogenetics, Leiden University Medical Center, Leiden 2333 ZC, The Netherlands and ^2^Division of Gene Regulation, The Netherlands Cancer Institute, Amsterdam 1066 CX, The Netherlands

## Abstract

Here, we report the investigation of microsatellite instability (MSI) in human cells with a newly developed reporter system based on fluorescence. We composed a vector into which microsatellites of different lengths and nucleotide composition can be introduced between a functional copy of the fluorescent protein mCherry and an out-of-frame copy of EGFP; *in vivo* frameshifting will lead to EGFP expression, which can be quantified by fluorescence activated cell sorting (FACS). Via targeted recombineering, single copy reporters were introduced in HEK293 and MCF-7 cells. We found predominantly −1 and +1 base pair frameshifts, the levels of which are kept in tune by mismatch repair. We show that tract length and composition greatly influences MSI. In contrast, a tracts’ potential to form a G-quadruplex structure, its strand orientation or its transcriptional status is not affecting MSI. We further validated the functionality of the reporter system for screening microsatellite mutagenicity of compounds and for identifying modifiers of MSI: using a retroviral miRNA expression library, we identified miR-21, which targets MSH2, as a miRNA that induces MSI when overexpressed. Our data also provide proof of principle for the strategy of combining fluorescent reporters with next-generation sequencing technology to identify genetic factors in specific pathways.

## INTRODUCTION

The human genome is full of DNA repeats. One abundant class of repeats, making up for ∼3% of the human genome (1), are microsatellites, which are often defined as repetitive runs of DNA sequences consisting of 1–8 bp long units (2). Soon after their discovery in the early 1980s, it became apparent that these tandem repeats are highly polymorphic in length and have mutation rates even up to 10^−^^2^ per locus per generation (3). It is their repetitive nature that makes microsatellites prone to mutagenesis; owing to strand slippage during DNA replication or unequal recombination, microsatellites can expand or contract. Microsatellites can be found all over the genome, present even in protein-coding sequences (4). Seventeen percent of human genes contain tandem repeats in their open reading frames (ORFs) (5), and microsatellites have been shown to affect biological processes such as chromatin organization, recombination, DNA replication, transcription and translation [reviewed in (6)]. It is therefore of no surprise that microsatellites are thought to play a significant role in evolution, and that many diseases, including several neurodegenerative diseases, and cancer are linked to variations in the length of genomic microsatellites.

The stability of microsatellites is influenced by several factors. An important factor is the status of Mismatch Repair (MMR). This pathway is well-conserved among species and consists of a delicate interplay of many proteins [for a review see for example (7) and references therein]. In brief, mis-incorporated nucleotides or small insertion–deletions loops are recognized by a heterodimeric protein complex consisting of MSH2 and MSH3 or MSH6. These mutS complexes interact with the mutL proteins MLH1 and PMS2, which are essential for incision and subsequent removal by EXO1 of the newly synthesized DNA. Numerous other proteins (e.g. PCNA, RFC, polymerase-δ, RPA and DNA ligase I) are required to complete the faithful repair of a mismatch or loop. Another important determinant that affects the stability of microsatellites is the length (the number of repeat-units) of the tract. Although a correlation between the length of the microsatellite and the mutation rate has been seen in numerous organisms (8–14), thus far there is no consensus whether this is a linear, quadratic or exponential relationship (10,15,16). Also, the genomic environment of the microsatellite is an important determinant for microsatellite instability (MSI): ample evidence exists that the locus where the microsatellite is situated is greatly affecting its stability (17–20). For example, a recent report showed that the presence of other repeats in close proximity of a microsatellite decreases its stability (20). Other factors like nucleotide composition, possible formation of secondary structures such as G-quadruplex structures and levels of transcription of the locus have also been implicated in the stability of microsatellites [as reviewed in (16)].

Many aspects on microsatellite dynamics have been studied in a plethora of organisms. However, several aspects have not been addressed in human cells, despite the notion that microsatellite dynamics clearly vary between organisms (even between humans and chimpanzees) (21). To gain full insight into MSI in human cells, we developed an experimental setup that is able to quantify MSI in human cells. We monitor MSI using a modular fluorescent reporter system in combination with fluorescence activated cell sorting (FACS). To exclude the influence of the genomic environment, we targeted different microsatellites to the same genomic locus. We addressed the influence of length, orientation, nucleotide composition, secondary structure, the transcriptional status of the locus as well as compound exposure. In addition, we show how this system aids to identify and characterize genetic regulators of MSI by assaying ∼450 miRNAs. This methodology can be easily adapted to read out other genome instability phenotypes in mammalian cells to uncover novel regulators in a specific pathway.

## MATERIALS AND METHODS

### Plasmid construction and sequencing

Standard molecular cloning techniques were used to obtain the constructs described in this manuscript. Briefly, using PCR, we amplified three DNA fragments: mCherry (from plasmid pRSET-B mCherry) without termination codon, flanked by a NheI and a HindIII restriction-site, a coding stuffer fragment of 215 bp flanked by a BamHI and an EcoRI restriction site, EGFP without start-codon flanked by EcoRI and EcoRV restriction sites (from pEGFP-N2, Clontech). Pieces were sequentially cloned into plasmid pcDNA5/FRT/TO (Life Technologies). Microsatellites were subsequently placed into the HindIII and BamHI site using oligo cloning. All plasmids sequences were checked by Sanger sequencing according to standard procedures, but with the following adjustments: we used a 1:3 mix of ABI Prism dGTP BigDye terminator v3.0 and BigDye terminator v3.0, 200 ng plasmid per sequencing reaction and no more than 25 cycles.

### Cell culture

Flp-in T-Rex-293 cells (Life Technologies) were cultured on poly-l-lysine coated surface at 37°C and 5% C0_2_ in DMEM (ref 41966, Gibco) supplemented with 10% fetal bovine serum (Bodinco BV) and 1% penicillin-streptomycin. Stable polyclonal Flp-in T-Rex 293 cell lines were generated via integration of the plasmids using Flp recombinase-mediated DNA recombination according to manufacturer’s protocol. Cells were cultured in the presence of 100 µg/ml hygromycin (ref 10843555001, Roche) and 15 µg/ml blasticidin (ref A11139-03, Gibco) and 0.1 µg/ml doxycycline hyclate (ref D9891, Sigma). MCF-7 cells with a single copy integrated pFRT/*lac*Zeo and the murine ecotrophic receptor were cultured under similar conditions, however selected with hygromycin and neomycin.

### Transient knockdown of MSH2

In all, 150.000 cells were seeded per well (12-well plate) per condition. Cells were transfected with a mix of 4 µl DharmaFECT1, 5 µl 20 µM ONTARGETplus smartpool MSH2 siRNA or non-targeting siGENOME Control pool (REF T-2001-03, L-003909-00, D-001206-13-05, Thermo Scientific Dharmacon) and 200 µl optimem after 24 h. Forty-eight hours later, cells were sorted, and 25.000 mCherry+EGFP− cells per well were seeded in a 96-well plate. A second round of knockdown was performed 24 h later, and cells were analysed 6 days later.

### Fragment analysis

mCherry−EGFP− cells were isolated from HEK 293 cells carrying plm405 (G_14_-repeat) by FACS. Forty-eight hours later, flow cytometry was used to sort one cell per well in a 96-well plate. Cells were grown to large colonies and checked by eye for expression of mCherry and EGFP. Next, DNA was isolated by NaCl/EtOH precipitation from single-cell colonies (mCherry+EGFP+, mCherry+EGFP−, mCherry−EGFP−). A standard PCR was performed using GoTaq DNA polymerase (REF M3175, Promega) and oligos CAGTCATAGCCGAATAGCCTCT and 6-FAM-labeled GACCACCTACAAGGCCAAGA. Samples were run on an ABI 3730 analyser (Applied Biosystems) and analysed with Peak Scanner software v1.0.

### Tests for transcription, ICR191 and Phen-DC6

#### Transcription

Cells were cultured in medium containing 0.1 µg/ml doxycycline hyclate and charcoal-stripped fetal bovine serum (ref A15-119, PAA laboratories). At day 1, mCherry+EGFP− cells with plm212 (C_23_-repeat) were sorted and cultured in doxycycline hyclate free medium. At day 3, a similar sort was performed, but now four cells per well were plated in a 96-well plate with or without doxycycline hyclate. At day 25, when wells were confluent, doxycycline hyclate was added to all plates. Cells were analysed for mCherry and EGFP expression by flow cytometry after 3 days.

#### Phen-DC_6_

Five thousand mCherry+GFP− cells of a polyclonal cell line with plm273 (G_11_AG_11_) were plated per well (96 well) and incubated with 5 µM Phen-DC_6_ (kindly provided by M.P. Teulade-Fichou, C Guetta and A. Nicolas) or control (DMSO). Cells were quantified by flow cytometry after 6 days.

#### ICR191

Five thousand mCherry−GFP− cells (with plm315, G_20_-repeat) were plated per well (96-well plate). Twenty-four hours later, cells were incubated with various concentrations of ICR 191(ref I3636, Sigma) dissolved in 0.01 MHCL and optimem for 2 h. Cells were analysed by flow cytometry after 9 days.

### miRNA screen

An MCF-7 reporter cell line was created by single copy integration of plm191 (G_23_-repeat). Next, a miRNA expression library (miR-Lib) (22) was used to transduce the reporter cell line with ±450 different miR-Vecs by retroviral transduction in a 96-well format in triplicate. Per experiment, cells were drug selected for 5 days, and all stable cell lines with integrated miR-Vecs were combined in one large pool. Next, mCherry+EGFP− cells were selected by flow cytometry, and after 21 days, mCherry+EGFP+ and mCherry+EGFP− populations were sorted per pool and genomic DNA was isolated (DNeasy kit, Qiagen). The miRNA coding regions from the retroviral (genomically integrated) miR-Vecs were amplified using IlluSeq_IndXX_Mirvec_f and P7_MirVec_r primers, followed by a second amplification step using P5_illuseq and P7_MirVec_r primers (sequences can be found in Supplementary Table S1). The resulting library was deep sequenced on an Illumina Hiseq platform according to manufacturers protocol. The resulting reads were aligned to the miR-lib resulting in 2.5 million aligned reads divided over six barcodes (triplicate measurements of two cell populations).

### Flow cytometry

Cells were sorted with flow cytometer BD FACSAria III and analysed with flow cytometers BD FacsDiva (BD biosciences) and guava easyCyte HT (Millipore) and their respective software. To set gates, we used control polyclonal cell lines that consisted out of solely mCherry+EGFP− or mCherry+EGFP+ cells.

## RESULTS AND DISCUSSION

### Construction and validation of a fluorescent microsatellite reporter

To study which factors contribute to the stability of microsatellites in human cells, we wished to develop a fast and reliable system that measures MSI independent of its genomic context. Therefore, we decided to make use of FACS enabling measurements of 10^6^–10^7^ events per hour and site-specific recombination to target microsatellites at the same specific locus, which ensures the identical genomic environment for each fragile tract, and thus complements and further extends previously described MSI-reporters that require episomal replication or random integration (23–25).

**Figure 1.**
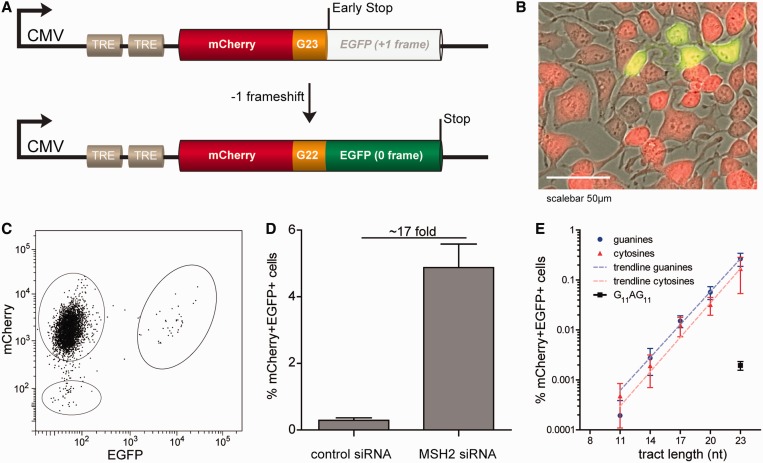
A fluorescent-based MSI reporter system at a fixed position in the human genome. (**A**) Schematic representation of the reporter transgene: the coding sequence of EGFP is placed in the +1 frame downstream of 23 guanines (G23) and the coding sequence of mCherry. A −1 frameshift brings the EGFP ORF in frame with the upstream mCherry. As a result, cells will express both markers. TRE = Tetracycline responsive element. (**B**) A representative image of an MSI event. In cultures, MSI events will manifest as single cells or a small clonal group of cells. Notably, mCherry+EGFP+ cells showed reduced expression levels of mCherry compared with mCherry+EGFP− cells. This image is an overlay of pictures taken in bright-field, green and red channel. (**C**) Representative FACS-plot of a population Flp-In T-Rex-293 cells with an integrated copy of the reporter transgene. The vast majority are mCherry+EGFP−, but also mCherry+EGFP+ are seen. (**D**) Knockdown of MSH2 by siRNA results in increased numbers of mCherry+EGFP+ cells (7 days after pre-sorting mCherry+EGFP− cells). Error bars represent the standard error of the mean (s.e.m.) of three experiments. (**E**) Exponential relationship between the number of nucleotides in a microsatellite (*X*-axis) and the incidence of frameshifting, as measured by the percentage of mCherry+EGFP+ cells (*Y*-axis) 2 days after pre-sorting mCherry+EGFP- cells. Values represent the means and s.e.m. of three independent experiments.

Accordingly, we created a transgene reporting MSI by expression of the green fluorescent protein EGFP on a frameshift; we placed the sequence of EGFP downstream of a microsatellite containing ORF such that a −1 bp frameshift at the microsatellite leads to in frame EGFP. The upstream ORF encodes functional red fluorescent protein mCherry (illustrated in Figure 1A). mCherry expression visualizes the presence of the reporter as well as the transcriptional activity of the transgene. Unique restriction sites flanking the microsatellite allow for easy insertion of different fragile sites. The sequence of this dual-fluorescent fusion product was cloned into the vector pcDNA5/FRT/TO (LifeTechnologies) that allows tetracycline-inducible expression under the control of a cytomegalovirus promotor (26–30). The vector also contains a single FLP Recombination Target (FRT) site that is required for Flp recombinase-mediated integration of the vector into a genome that contains a single copy integrated FRT site (31,32). This system allowed us to efficiently create stable cell lines with a single copy integrated reporter containing a microsatellite.

First, we tested a reporter containing a microsatellite of 23 guanines in the coding strand (plm191, Figure 1A) in Flp-In T-REX-293 cells that contain a single copy integrated Flp Recombination Target site (LifeTechnologies). As expected, the majority of cells expressed mCherry but not EGFP (mCherry+EGFP−) after integration of the reporter; however, a small percentage (±0.2%) also expressed EGFP (mCherry+EGFP+), as visualized by microscopy and FACS (Figure 1B and C). As a control, we integrated a similar reporter but without a microsatellite (plm184). This cell line showed only mCherry+GFP− cells (data not shown). Together, this indicated that EGFP expression was the consequence of the presence of the microsatellite. To further substantiate that the EGFP+ cells were the result of MSI, we performed a transient knockdown of the MMR-factor MSH2 by siRNA on pre-sorted mCherry+EGFP− cells. Because the MSH2 protein was reported to be stable (33), two rounds of siRNA-treatment were performed, and FACS analysis was performed after 7 days of culturing. This resulted in a ±17-fold increase in the number of mCherry+EGFP+ cells, indicative for MSI-dependent expression of EGFP (Figure 1D). Together, these data indicate that EGFP expression is a read-out for MSI. The advantage of the mCherry internal control lies in the fact that by pre-sorting mCherry+EGFP− cells, we can (i) discard cells that became EGFP positive before an experimental condition was applied and (ii) get rid of cells that lost the reporter or its expression due to silencing.

### Exponential correlation between length of a monotract and MSI

To determine the type of correlation between the length of the microsatellite tract and its instability, we made transgenes carrying monotracts of lengths 8, 11, 14, 17, 20 and 23 nt, of all four different nucleotides. On integration in 293-T-REX cells, stable polyclonal lines were obtained. Unexpectedly, we found that adenine and thymine monotracts diminished EGFP expression levels (when put in frame as a control), making MSI analysis of these tracts impossible. Therefore, we pursued analysis with the lines containing monotracts of guanines and cytosines. We established populations of 100% mCherry+EGFP− cells (by FACS) and determined the percentages of mCherry+EGFP+ after 48 h of culturing. We started to detect MSI in cell lines containing monotracts of 11 bp or longer; in cells with monotracts of 8 nt (either guanine or cytosine), no mCherry+EGFP+ cells were detected, corresponding to a mutation frequency that is <10^−^^5^. This result may be explained by the inability of a small monotract to induce slippage events: several *in vitro* and computational studies have argued a threshold for slippage being ∼7–9 bp (15,34–37). Figure 1E indicates that the correlation of MSI and the length of a monotract is best fitted by an exponential trend line; for every extra guanine or cytosine in the monotract, the number of mCherry+EGFP+ cells increased with 1.66- and 1.70-fold, respectively (R^2^ = 0.80 and 0.43). We found no significant difference in MSI between cell lines containing similar sized guanine or cytosine monotracts, suggesting no influence of strand orientation, similar to what was reported in yeast (38).

### Interruption of pure repeats

To explain the distribution and stability of microsatellites across genomes, several groups have postulated mutational models [reviewed in (16)]. Experimental evidence, as described earlier in the text, helps to improve these mutational models. Rates for expansions and contractions are obviously important parameters in these models; however, another factor of great influence is the effect of point mutations that split up a monotract into two smaller ones. To measure the effect of such an event experimentally, we analysed a reporter (plm273) with an instability locus that is similar to a G23 monotract, apart from having an A instead of a G at position 12, hence comprising two monotracts of 11 Gs (G_11_AG_11_). We found that the MSI-rate of this fragile site was >100-fold lower than that of a G23 monotract (Figure 1E). These results show that a single mutation can lead to stabilization of a repeat with more than two orders of magnitude. Similar observations were found in human MMR-deficient cells (39). Interestingly, the G_11_AG_11_ repeat was still 10-fold more unstable than a single G11 repeat, suggesting that two repeats in close proximity are more unstable than the sum of two single G11 repeats.

### Contraction exceeds expansion of longer monotracts

During the course of our experiments, we noticed the presence of mCherry−EGFP− cells by microscopy and FACS (Figure 1C, lower gate). These cells were resistant to the transgene encoded selection-marker, which is located directly upstream of the fluorescent reporter, making silencing or loss of the locus unlikely. Interestingly, we noticed that the fraction of mCherry−EGFP− cells was similar to the fraction of mCherry+EGFP+ cells. We thus wondered whether mCherry−EGFP− cells are the result of +1 frameshift events: although the monotracts were cloned downstream of mCherry to not interfere with its expression, it may be that the −1 frame of GFP encodes an amino acid sequence that interferes with proper mCherry expression.

**Figure 2.**
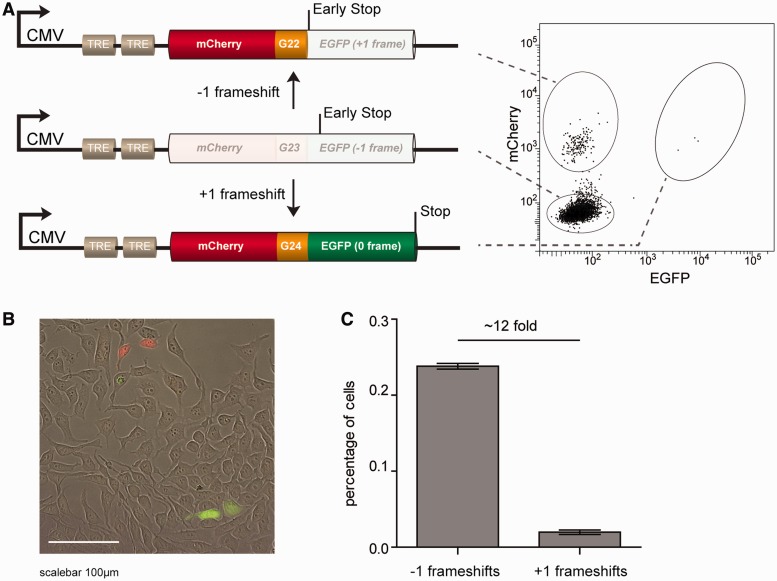
A fluorescent-based reporter system that reads out both −1 and +1 frameshifts (**A**) Schematic representation of the reporter construct. When the coding sequence of EGFP is placed in the −1 frame neither mCherry nor EGFP is expressed. A −1 frameshift results in mCherry+EGFP− cells, whereas a +1 frameshift results in mCherry+EGFP+ cells. The right panel illustrates the three different populations in a representative FACS-plot. (**B**). Representative image of HEK293 cells carrying a single copy integrated reporter transgene. (**C**) Quantification of percentages mCherry+EGFP− and mCherry+EGFP+ cells representing −1 and +1 frameshifts, respectively, 2 days after pre-sorting mCherry-EGFP- cells. Error bars denote standard deviation (s.d.) of three independent experiments.

To test this explanation, we constructed transgenes with EGFP in the −1 reading frame. Indeed, cells with integration of such constructs resulted in three populations of cells: the vast majority of cells being mCherry−GFP−, and two smaller populations of mCherry+EGFP− and mCherry+EGFP+ cells (Figure 2A). To confirm that the latter two populations represented −1 and +1 bp frameshifts, respectively, we performed fragment analysis on these populations. A cell line containing a G14 monotract was used because analysis of long repeats is technically challenging. Indeed, fragment analysis on colonies derived from single cells confirmed that mCherry+EGFP− represented −1 frameshifts, whereas mCherry+EGFP+ represented +1 frameshifts (see Supplementary Figure S1). We thus serendipitously constructed a transgenic reporter that allows the quantification of both contractions and expansions of microsatellites.

Subsequently, we used this experimental setup to investigate whether −1 and +1 events are induced with equal frequencies. We isolated mCherry−EGFP− cells of transgenes containing a G_23_ monotract and analysed the populations 48 h later. We found 12 times more mCherry+EGFP− cells than mCherry+EGFP+, indicating that 1 bp contractions are more prevalent than 1 bp expansions. This result supports studies reporting that longer microsatellites are more prone to contracting (12,40).

### No role for on-going transcription on MSI levels

**Figure 3.**
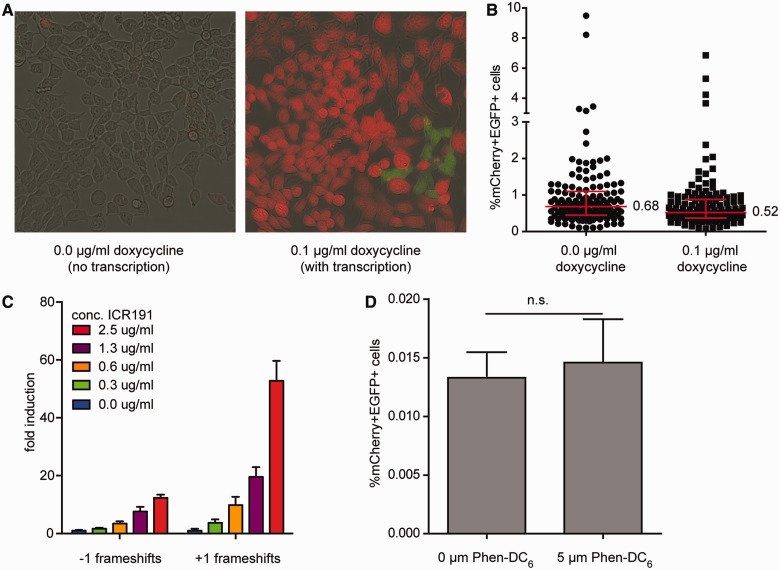
Structural and chemical determinants of MSI. (**A**) Representative images of HEK293 cells carrying a single copy C23 reporter transgene in the absence or presence of doxycycline. Images are overlay of pictures taken in bright-field, green and red channel. (**B**) Percentage of mCherry+EGFP+ HEK293 cells carrying a single copy C23 reporter after 28 days of culturing. Three days before flow cytometry, doxycycline was added to all cells to read out reporter expression. The graph represents the data of ∼140 individually grown populations per condition derived from three independent experiments. Error bars denote the median with interquartile range. Percentage of median is shown. (**C**) mCherry-GFP- HEK 293 cells carrying a single copy G_20_ reporter, that reads out −1 and +1 events, were treated with the indicated concentrations of ICR191 for 2 h and analysed by flow cytometry 9 days later. The data represent eight replicates derived from two independent experiments. Error bars denote the s.e.m. (**D**) mCherry+EGFP− HEK 293 cells containing a single copy G_11_aG_11_ reporter were grown for 6 days in the presence or absence of the G4 stabilizer Phen-DC_6_ and analysed by flow cytometry. Data represent 32 replicates derived from two independent experiments. Error bars denote the s.e.m, n.s = not significant by unpaired two-tailed *t*-test.

Studies in bacteria and yeast have shown that transcription can destabilize simple repetitive DNA sequences (41,42). We questioned if and to which extent transcriptional activity affects MSI in human cells by monitoring frameshifting at monotracts in cells that were cultured in the presence or absence of doxycycline, a regulator of the Tet repressor that controls the levels of transcription over the transgene (43). To avoid transcriptional activity of the transgene at the time of pre-sorting, cells were grown in doxycycline-free medium for three days, resulting in low but still detectable levels of mCherry. Then, four single mCherry+EGFP- cells were seeded and the cultures were grown till confluency, while the transcriptional status of the transgene was visualized by mCherry expression (Figure 3A). To be able to use EGFP as a marker, doxycycline was also added to the non-transcribed conditions 3 days before reading out MSI. In contrast to studies in bacteria and yeast, MSI is not elevated by ongoing transcription in human cells (Figure 3B).

### MSI-reporter applicable for testing compounds

One of the applications of the system we developed resides in testing compounds for possible mutagenic characteristics in mammalian cells. As proof of principle, we used the compound 6-chloro-9-[3-2(2-chloro-ethylamino)propylamino]-2-methoxy-acridine (ICR191), an acridine derivate that is a known inducer of frameshift mutations (44). We exposed mCherry−EGFP− cells with a G_20_-repeat to various concentrations of ICR191 and allowed them to grow for several days, before analysis by flow cytometry. Figure 3C shows a dose-dependent increase of both −1 and +1 frameshifts. In addition, we observed, in line with previous studies (44–48), that +1 frameshifts were more frequently induced by ICR191 than −1 frameshifts (53- versus 12-fold, respectively).

### Quadruplex formation and MSI

Non-B-DNA conformations of microsatellites are thought to facilitate slippage and thus influence the frameshift mutation rate (16). One of such non-B-DNA structures is a G-quadruplex structure; a secondary structure that consists of minimally three stacked planar arrays of four guanines held together by Hoogsteen hydrogen bonding (49). In this study, we have used several microsatellites that have the ability to form a G-quadruplex structure. To address a possible influence of G-quadruplex formation on MSI, we assayed cells containing a G_11_AG_11_-repeat, a sequence that has the potential to form a G4-quadruplex structure *in vivo* (Koole *et al.*, unpublished), in the presence and absence of Phen-DC_6_ (50) an effective stabilizer of G4-structures in yeast and human cells (51–54). We found, however, similar level of MSI in cells treated or mock-treated with to Phen-DC_6_ (Figure 3C), suggesting that quadruplex formation does not stimulate frameshift mutations in monotracts.

### Role of miRNAs on MSI

**Figure 4.**
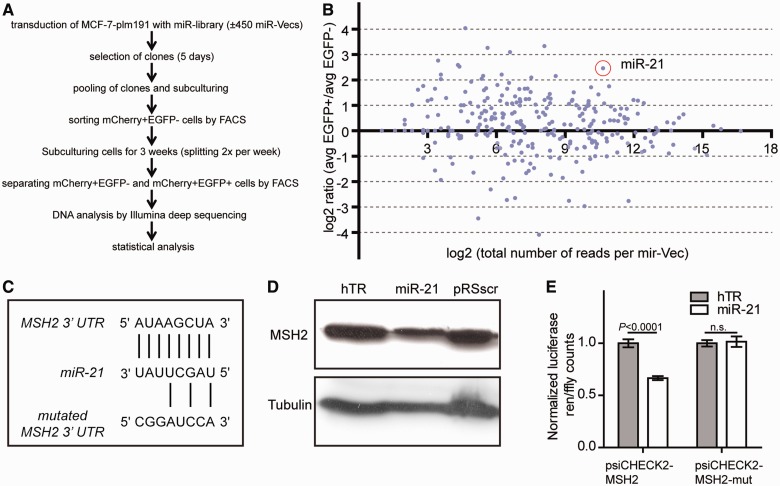
An unbiased screen for miRNAs affecting MSI reveals miR-21 as negative regulator by affecting MSH2 levels. (**A**) Flow chart of the micro-RNA screen using MCF-7 cells that contain a single copy integrated MSI reporter. (**B**) A dot plot of the relative abundance of miR-Vec constructs in cells that were selected for an MSI event. The *X*-axis represents the Log2 count per miR-Vec, as quantified by deep sequencing. The *Y*-axis represents the Log2 ratio of the relative abundance of a miR-Vec in EGFP(+) over EGFP(−) cells. (**C**) Predicted conserved binding site in 3′ UTR of *MSH2* for miR-21. The seed sequence of miR-21 is shown (1–8 bp). Lower sequence illustrates the mutated version of the 3′ UTR of *MSH2* that was used for psiCHECK2-MSH2-mut. (**D**) Immuno-detection of MSH2 protein levels in MCF-7 cells with integrated miR-Vec miR-21 or control vectors hTR or pRSscr. Tubulin was stained to control for protein loading. (**E**) Dual-luciferase-assay (Promega) using vectors psiCHECK2-MSH2 and psiCHECK2-MSH2-mut in combination with co-transfection of miR-21 or control plasmid (hTR). Error bars denote s.d., statistical analysis: unpaired two-tailed *t*-test.

Besides testing intrinsic factors, such as the sequence context of microsatellites, and exogenous influences, such as mutagenic compounds, the reporter system also allows systematic testing for genetic factors and their effect on MSI (as demonstrated by siRNA knockdown of MSH2). Moreover, also unbiased screens are possible to identify genetic regulators of MSI: as proof of principle, we used an unbiased screening approach to identify miRNAs that influence MSI (Figure 4A). To this end, we integrated a single copy of a transgene containing a G_23_-repeat (plm191) into a human breast carcinoma cell line MCF-7. The resulting cell line was transduced with a miRNA expression library consisting of expression constructs (miR-Vecs) for most human miRNAs (22). Stable cell lines with integrated miR-Vecs were pooled and mCherry+EGFP− cells were selected by flow cytometry to remove cells with mutation events that occurred during the transduction and selection process. After 3 weeks of culturing, mCherry+EGFP− and mCherry+EGFP+ cells were sorted, and genomic DNA was extracted and subjected to next-generation sequencing to determine the relative abundance of each miRNA insert per population. Of a total of 301 data points for individual miR-Vecs (Figure 4B), we selected 17 miRNAs for independent validation experiments. Of these 17 miRNAs, only miR-21 reproducibly induced MSI. Cell growth and plating efficiency was not affected by miR-21 overexpression (data not shown). Several target genes have been described for miR-21, including the MMR gene MSH2 (55). The microRNA binding prediction program targetscan predicts a binding site for miR-21 in the 3′UTR of human *MSH2* mRNA (Figure 4C). Using immunoblotting experiments, we observed a visible reduction of MSH2 protein levels after miR-21 overexpression (Figure 4D) arguing that MSH2 is a *bona fide* target of miR-21. To further substantiate this interaction, we cloned the *MSH2* 3′UTR downstream of the Renilla luciferase gene. Co-transfection of miR-21 with this reporter construct (psiCHECK2-MSH2) in HEK 293 cells [which have low miR-21 levels (56)] led to a ±40% reduction in luciferase counts, supporting the notion of negative regulation of MSH2 by miR-21 (Figure 4D). To demonstrate the specificity of miR-21 in targeting the 3′UTR of MSH2, we mutated its binding site by site-directed mutagenesis (Figure 4C), resulting in reporter construct psiCHECK2-MSH2-mut. These mutations killed the downregulatory effect of miR-21 overexpression on luciferase expression (Figure 4E), thus providing proof for specificity in miR-21’s genetic interaction with the evolutionary conserved binding site in the 3′UTR of MSH2. These results also provide proof of principle that our newly developed reporter system can be used to find (novel) *bona fide* genetic modifiers of MSI.

## CONCLUSION

In this study, we describe a functional fluorescence-based reporter that reads out MSI *in vivo* at one specific locus and can be measured by flow cytometry allowing for high-throughput analysis. To prove the functionality of this reporter and meanwhile obtaining biological informative data, we tested several repeat-intrinsic and environmental factors that can influence MSI in human cells. In addition, candidate genes can be screened for factors that are potentially implicated in MSI. We combined retroviral transduction of several hundred miRNA-overexpressing constructs with next generation sequencing to identify a regulator of MSI when overexpressed. Although we made use of a miRNA-library and an MSI-specific reporter, we would like to point out that this approach is applicable to any other type of library (e.g. compound or knockdown libraries) and is easily adaptable to readout other potential fragile sequences or other specific pathways when using adjusted fluorescent reporters.

## SUPPLEMENTARY DATA

Supplementary Data are available at NAR Online.

## FUNDING

European Research Council [203379, DSBrepair, to M.T.]; European Commission (DDR Response); Zorg Onderzoek Nederland/Medische Wetenschappen/Netherlands Genomics Initiative-Horizon (to M.T.); Koningin Wilhelmina Fonds [Hubr2008-4107 to M.T.]; VENI-NWO (Nederlandse Organisatie voor Wetenschappelijk Onderzoek) (to G.v.H.); Forschungsstipendium der Deutsche Forschungsgemeinschaft (to H.S.S.). Funding for open access charge: European Research Council.

*Conflict of interest statement*. None declared.

## Supplementary Material

Supplementary Data
