# Supplementary material for: A versatile microsatellite instability reporter system in human cells
Source: Nucleic Acids Res. 2013 Jul 16;41(16):e158. doi: 10.1093/nar/gkt615 (PMC3763563; doi:10.1093/nar/gkt615)
Supplement: Supplementary Data [file supp_41_16_e158__index.html]

A versatile microsatellite instability reporter system in human cells — A versatile microsatellite instability reporter system in human cells — Supplementary Data 

# A versatile microsatellite instability reporter system in human cells

## 

files

**Files in this Data Supplement:**

- Supplementary Data - pdf file
