# Supplementary material for: A versatile microsatellite instability reporter system in human cells
Source: Nucleic Acids Res. 2013 Jul 16;41(16):e158. doi: 10.1093/nar/gkt615 (PMC3763563; doi:10.1093/nar/gkt615)
Supplement: Supplementary Data [file supp_gkt615_nar-01415-met-k-2013-File006.pdf]

Supplementary table 1 Primers used for amplification of miR-Vecs

Primer	Sequence
IlluSeq_Ind01_MirVec_f	ACACTCTTTCCCTACACGACGCTCTTCCGATCTaaGCTTGGTACCGAGCTCGGATC
IlluSeq_Ind02_MirVec_f	ACACTCTTTCCCTACACGACGCTCTTCCGATCTatGCTTGGTACCGAGCTCGGATC
IlluSeq_Ind03_MirVec_f	ACACTCTTTCCCTACACGACGCTCTTCCGATCTagGCTTGGTACCGAGCTCGGATC
IlluSeq_Ind04_MirVec_f	ACACTCTTTCCCTACACGACGCTCTTCCGATCTacGCTTGGTACCGAGCTCGGATC
IlluSeq_Ind05_MirVec_f	ACACTCTTTCCCTACACGACGCTCTTCCGATCTtaGCTTGGTACCGAGCTCGGATC
IlluSeq_Ind06_MirVec_f	ACACTCTTTCCCTACACGACGCTCTTCCGATCTttGCTTGGTACCGAGCTCGGATC
IlluSeq_Ind07_MirVec_f	ACACTCTTTCCCTACACGACGCTCTTCCGATCTtgGCTTGGTACCGAGCTCGGATC
IlluSeq_Ind08_MirVec_f	ACACTCTTTCCCTACACGACGCTCTTCCGATCTtcGCTTGGTACCGAGCTCGGATC
IlluSeq_Ind09_MirVec_f	ACACTCTTTCCCTACACGACGCTCTTCCGATCTgaGCTTGGTACCGAGCTCGGATC
IlluSeq_Ind10_MirVec_f	ACACTCTTTCCCTACACGACGCTCTTCCGATCTgtGCTTGGTACCGAGCTCGGATC
IlluSeq_Ind11_MirVec_f	ACACTCTTTCCCTACACGACGCTCTTCCGATCTggGCTTGGTACCGAGCTCGGATC
IlluSeq_Ind12_MirVec_f	ACACTCTTTCCCTACACGACGCTCTTCCGATCTgcGCTTGGTACCGAGCTCGGATC
P5_IlluSeq	AATGATACGGCGACCACCGAGATCTACACTCTTTCCCTACACGACGCTCTTCCGATCT
P7_MirVec_r	CAAGCAGAAGACGGCATACGAGATAACCTACAGGTGGGGTCTTTC
P7	CAAGCAGAAGACGGCATACGAGAT
P5	AATGATACGGCGACCACCGAGATCT

Supplementary Figure 1

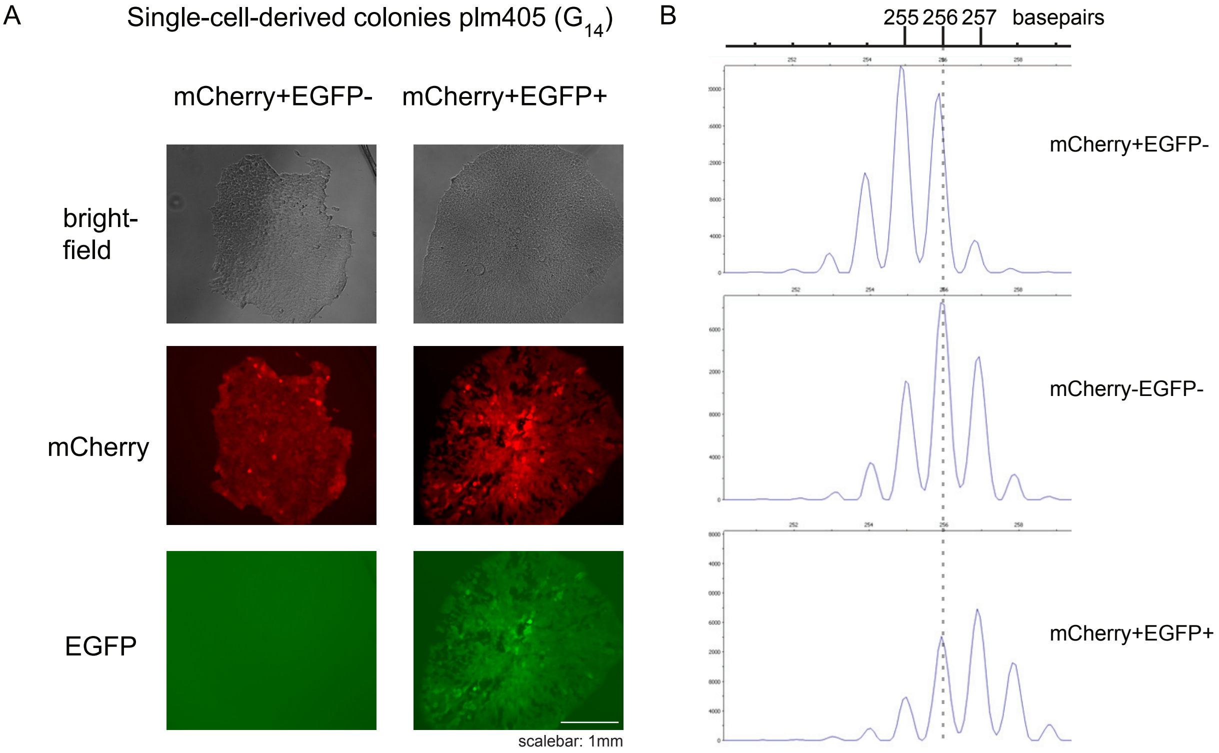

Fragment analysis of mCherry+EGFP- and mCherry+EGFP+ expressing cells. (A) Images of single cell derived colonies from HEK 293 cells with a G_{14} reporter. Images represent an mCherry+EGFP- (left) and an mCherry+EGFP+ colony (right). (B) Representative images of DNA fragment analysis on single cell-derived colonies. The highest peak represents the length of the amplified PCR-product that includes the microsatellite. Fragment analysis revealed that 8/9 mCherry+EGFP- colonies were the result of -1 events, whereas 26/27 mCherry+EGFP+ colonies suffered from a +1 event.
